# Schistosome Vaccine Adjuvants in Preclinical and Clinical Research

**DOI:** 10.3390/vaccines2030654

**Published:** 2014-09-02

**Authors:** Rachel Stephenson, Hong You, Donald McManus, Istvan Toth

**Affiliations:** 1 School of Chemistry and Molecular Biosciences, The University of Queensland, St. Lucia, QLD 4072, Australia; E-Mail: i.toth@uq.edu.au; 2 Molecular Parasitology Laboratory, Infectious Diseases Division, QIMR Berghofer Medical Research Institute, Brisbane, Queensland 4101, Australia; E-Mails: Hong.You@qimrberghofer.edu.au (H.Y.); Don.McManus@qimrberghofer.edu.au (D.M.); 3 School of Pharmacy, The University of Queensland, Woolloongabba, QLD 4102, Australia

**Keywords:** adjuvant, vaccine, helminth, schistosoma

## Abstract

There is currently no vaccine available for human use for any parasitic infections, including the helminth disease, schistosomiasis. Despite many researchers working towards this goal, one of the focuses has been on identifying new antigenic targets. The bar to achieve protective efficacy in humans was set at a consistent induction of 40% protection or better by the World Health Organisation (WHO), and although this is a modest goal, it is yet to be reached with the six most promising schistosomiasis vaccine candidates (Sm28GST, IrV5, Sm14, paramyosin, TPI, and Sm23). Adjuvant selection has a large impact on the effectiveness of the vaccine, and the use of adjuvants to aid in the stimulation of the immune system is a critical step and a major variable affecting vaccine development. In addition to a comprehensive understanding of the immune system, level of protection and the desired immune response required, there is also a need for a standardised and effective adjuvant formulation. This review summarises the status of adjuvants that have been or are being employed in schistosomiasis vaccine development focusing on immunisation outcomes at preclinical and clinical stages.

##  1. Introduction

Schistosomiasis, also called bilharzia having been first described by Theodor Bilharz over 150 years ago, is a blood-dwelling trematode fluke worm. With approximately 200 million people infected in over 74 countries, schistosomiasis is recognised as the most important human helminth infection in terms of morbidity and mortality [1,2]. Despite over 20 years of highly effective chemotherapeutic (praziquantel) drug treatment integrated with improved sanitation and hygiene measures, this disease is still spreading into new areas of the globe [1,3]. Limitations of current treatment regimes, which include high rates of reinfection, the potential development of drug-resistant parasites, the effective administration of drugs requiring a large infrastructure to cover all parts of an area of endemicity, and the associated costs have further supported research for an effective vaccine strategy to complement current treatment for future control and possible elimination of this parasitic disease [1,3].

Schistosomiasis immunology has provoked considerable research interest over the past 30 years with many questions remaining unanswered. These relate to the development of the many pathological changes that accompany the infection, how some infected individuals can develop resistance to infection, and lastly, that schistosome worms can survive in the mammalian host for many years despite a strong immune response being generated [4].

Despite intensive research, identification and development of suitable anti-schistosome vaccine candidates has been slow [5]; however, an improved understanding of the immune response to *Schistosoma* infection in both human and animal models suggests that vaccine development is possible [3]. The development of schistosomiasis vaccines can be assigned to three types: (1) a prophylactic vaccine aimed at preventing or reducing infection and indirectly transmission and/or leaving no worms in the host; (2) a vaccine aimed to reduce or eliminate reinfection intensity or transmission by interrupting female worm survival or egg production; or (3) a therapeutic vaccine to reduce disease but not affect infection or transmission [5]. One reason for the slow progress in developing an effective schistosome vaccine is the strong capacity of schistosome parasites to evade a host’s immune response. This arises, in part, from the pathogen’s complexity and its ability to exhibit genetic diversity as well as antigenic variation during the multistage life cycle. Subsequently, to achieve host protection against schistosomiasis, an immune response combining both humoral and cellular responses that target different stages of the parasites life cycle are essential [6].

**Figure 1:**
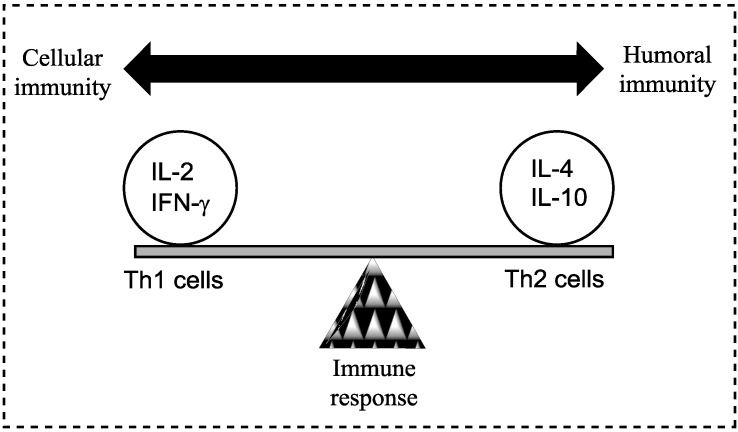
A model demonstrating the balance between Th1 (cell-mediated) and Th2 (humoral) response of the adaptive (specific) immune system; a balance is essential to prevent disease. Both Th1 and Th2 responses are tightly controlled but excessive activation may cause or alter the disease state. Cytokines are most commonly grouped by their functional similarities and one of the most prominent concepts used to discriminate two distinct ways that the specific immune system can react on environmental stimuli is the classification of Th1 and Th2 cell diversity. This classification is based on the cytokine production patterns of T helper cells and reflects the polarization of the immune system to either a cell-mediated (Thl) or a humoral (Th2) immune response [8].

Schistosomes appear to have evolved a number of strategies to down-regulate the host’s immune response to promote their own survival [7]. T cell mediated immunity has been shown to be essential in the fight against schistosomiasis (reviewed by [3,4,7,8] and references within). Schistosomes, like other parasitic helminths, induce prominent T helper type 2 (Th2, humoral) responses with a quantifiable shift from gamma interferon (IFN-γ) and T helper type 1 (Th1, cellular) responses to an elevated production of interleukin (IL)-4 and Th2 in the spleens of infected mice [4,9]. However, disease severity and the immune response is complicated by the host’s genetics, the intensity of the infection, co-infection status, and *in-utero* sensitisation to schistosome antigens (compared to a naïve individual) [4]. Furthermore, humans who are infected with schistosomes, in general, have a Th2 type response, but, on the basis of IFN-γ and IL-5 levels, a Th1-like immune response is generated for some individuals [4]. IL-10 has also been shown to play an important role in schistosomiasis by preventing the development of Th1 and Th2-mediated pathologies [4]. Overall, a balance between Th1 and Th2 is required and a skewed response too heavily in either direction has harmful consequences for the host. Indeed, the vast majority of people living with schistosome infections elicit a balanced immune response that holds both the pathology and parasite in harmony (Figure 1) [8].

The initial trigger for the Th2 response is unclear and complicated by the different developmental stages of the schistosome parasite. The deposition and entrapment of eggs in the liver, spleen and lungs tissues elicits a Th1 immune response and characterised by the production of IL-12, IFN-γ and tumour necrosis factor (TNF). The early immune response following infection with cercariae is predominantly a Th1 response, targeted at the adult worm [10]. As the transition of the disease takes place from mild to chronic, a shift to a Th2 response in observed with the production of anti-inflammatory cytokines (IL-4, -5, -10, and -13) and parasite-specific antibodies [8]. The inflammation and granuloma formation are thought to be responsible for the strong Th2 immune response, rather than the presence of the schistosomula or adult worms [11]. Additionally, the trapped eggs secrete a range of molecules leading to a marked MHC II CD4^+^ T cell inflammation characterised by eosinophils (whose role in the infection process remains undetermined), monocytes, and lymphocytes, in addition to collagen deposition and hepatic fibrosis [4]. The generic nature of the Th2 bias (where cytokine IL-4 is deemed essential for the polarisation of the Th2 response) during infection suggests the possibility that the vertebrate innate immune system recognises conserved parasite motifs, preferentially triggering a Th2 response [11]. Furthermore, fibrosis is inhibited in mice that are immunised with IL-12 and egg antigens, with cytokines (IFN-γ and TNF) present, preventing Th2 biasing and natural immunity [4]. It has been suggested that a vaccine activating macrophage-induced Th1 cytokines (IFN-γ and IL-2) may help in the prevention of schistosomiasis and, although the mechanism is unclear, an IgE antibody-dependent targeted cellular cytotoxicity has also been shown to protect humans. Additionally, further cytokines (*i.e.*, IL-1, TNF-α, IL-6) have been shown to have supporting roles in the host’s immune response and the mechanism of protection [3,4,7].

Immunisation is considered one of the most effective of the public health interventions. Despite the advantages of prevention over treatment whereby traditional vaccines evolved from a prophylactic role (prevention of disease), a large market exists for vaccines to treat diseases. However, to date, no therapeutic vaccines have been approved [12]. Elicitation of immunological memory in traditional vaccine-exposed individuals to whole live attenuated organisms or killed micro-organisms against which they were being immunised elicited a strong immune response, associated in part with the capacity of the pathogen to replicate, and to be retained at the administration site. However, disadvantages in this approach resulted in a great deal of research being focused on alternative vaccine development techniques [12,13]. Current methods of vaccine design use a subunit approach whereby only the minimal microbial components necessary to stimulate an appropriate immune response are incorporated into the vaccine; however, despite the potential advantages of subunit vaccines, poorly immunogenic vaccines are produced, necessitating administration with powerful adjuvants, and in some cases, the addition of T helper epitopes to elicit a lasting immune response [12].

An immunological adjuvant, when incorporated into a vaccine formulation, accelerates, prolongs or enhances the quality of specific immune responses to vaccine antigens [14]. The mechanism of action for adjuvants include: (1) increasing the biological or immunologic half-life of vaccine antigens; (2) improving antigen delivery to antigen-presenting cells (APCs), as well as antigen processing and presentation by the APCs; and (3) inducing the production of immunomodulatory cytokines. Through modulation of cytokine responses, adjuvant formulations can be designed that favour the development of Th1 or Th2 immune responses to vaccine antigens [14]. However, despite the important role of adjuvants, relatively few have been incorporated successfully into vaccines intended for human administration [12].

This review summarises adjuvants in preclinical and clinical *Schistosoma* research where adjuvants are evaluated for their role in effective vaccine development focusing on their pharmaceutical and immunological properties. Adjuvants can be classified by their sources, mechanisms of action or chemical properties. Table 1 lists the types of adjuvants under preclinical and clinical development for use with schistosome vaccines. Further, a description of *Schistosoma* antigen candidates cited in this review are summarised in Table 2. A compilation of *Schistosoma* vaccine candidates clinically tested and classified by adjuvant is presented in Table 3, Table 4, Table 5, Table 6, Table 7 and Table 8 [6].

## 2. Major Schistosome Vaccine Adjuvants under Preclinical and Clinical Evaluation

This section describes adjuvants that have been evaluated in preclinical and clinical trials of *Schistosoma* vaccine candidates, and considers the most recent and relevant studies.

### 2.1. Gel-Type

The first mechanism of adjuvant action identified was the depot effect in which gel-type adjuvants (such aluminium hydroxide) associated with the antigen and facilitated antigen transport to the draining lymph node, generating an immune response [14].

#### Alum

Alum is an insoluble gel-like precipitate derived from aluminium hydroxide or aluminium phosphate with a particle size from 100–1000 nm. This was the first adjuvant approved for human use approximately 80 years ago and has become the component of numerous licensed human and veterinary vaccines since 1930 and has an excellent safety record [6,15].

Alum has the capacity to stimulate strong humoral responses (Th2) although the interaction of Alum with the immune system is not well defined [9]. Alum was first believed to only produce a depot effect and thereby a sustained release of antigen, but several studies have reported a rapid desorption of this adjuvant from the injection site. Additionally, it is now clear that the administration of the antigen in a particulate form favours its capture by APCs [6] with strong IgE responses also being reported. Alum salts are inexpensive, safe and simple to formulate [15] but are generally weaker adjuvants than emulsion adjuvants [15].

Clinical testing of Alum-adjuvanted *Schistosoma* vaccines has resulted in a high reduction in the number of worms (66%–77% in primates) and eggs (66% in primates) (Table 3) in vaccinated animals. Additionally, recombinant *Schistosoma haematobium* (*S. haematobium*) 28-kD glutathione S-transferase (Sh28GST) protein formulated with Alum is the only *Schistosoma* vaccine (commercial name Bilhvax) in phase III clinical trials; although results of the trial were anticipated in late 2013, no data are yet available [5]. Previous phase I and II evaluations of this candidate demonstrated that the vaccine is safe in adults (healthy and infected persons) and children. The vaccine has been shown to be immunogenic as shown by induction of high IgG1, IgG2, IgG3 titres and the production of Th2 type cytokines (IL-5, IL-10, IL-13) [5].

**Table 1:** Types of immunological adjuvants.

Type	Adjuvant
Gel-Type	Alum
Emulsion	RIBI
	TiterMax
	FCA
	IFA
	GLA-SE
	Montanides
Particulate	Saponins
	Liposomes
	Polysaccharides/Oligosaccharides
	Synthetic polynucleotides
	Peptide analogues
	Imidazoquinolines
Cytokine	IL-12, IL-4, IL-18
	TSLP
Microbial	BCG
	CT
Protease	Papain

**Table 2:** Description of some *Schistosoma* antigen candidates.

Stage Expressed	Antigen Abbreviations	Description	References
Schistosomula, adults	Paramyosin (pmy)	Structural component of invertebrate muscle	[16,17]
All stages	Sm14, Sj14, SjFABP, Fh15FABP, Sj14-3-3	Fatty acid binding protein located below the sub-tegumental region of the male worm, and in the vitelline droplets of the vitelline glands of the female worm. The latter provides nutrients to the developing egg	[17,18]
Schistosomula, adults	Sm-tsp-1, Sm-tsp-2	Tetraspanin surface antigen containing B and T cell surface receptors, are abundant in both the lung-stage larval and adult stage parasites, and are crucial for the assembly and maintenance of protein scaffolds by which proteins are laterally organised	[6]
Adults	Sm22,6, Sm29	Tegument proteins	[18,19]
Adults	Sm-CatD	Epitope on surface of the cathepsin D hemoglobinase protein which plays a pivotal role in digestion of the blood fluke’s bloodmeal	[20]
Cercariae, Schistosomula, adults	Sjc-97 pmy, Sm97, Sj97	Myofibrillar protein found in the muscle layer	[6,21]
All stages	Sj26GST, Sb28GST, sm28GST, Sh28GST	Glutathione *S*-transferase enzyme, localised within the parenchymal region of the male parasite and in the parenchymal cells between the vitelline glands in the female worms, are a group of isoenzymes that catalyse the detoxification by thioconjugation of lipophilic molecules	[6,17,21,22]
All stages	Sj23, Sm23	Trans-membrane-4 superfamily integral membrane protein consisting of four hydrophobic trans-membrane domains and a large and small hydrophilic domain, both of which are thought to be extracellular	[6,21]
All stages	SjTPI, SmTPI	A dimeric enzyme which converts glyceraldehyde-3-phosphate to dehydroxyacetone phosphate, an important step in the glycolytic pathway, is located at the surface membrane of newly transformed schistosomula and present in cells (gut, muscles and the tegument) of the adult worm inhibiting glycolysis	[5,17,21]
All stages	Sm-p80	Calpain is a surface membrane protein which affects parasitic surface membrane renewal and aids in the recycling of the tegument from lung-stage schistosomula or epithelial syncytium of the adult parasite	[5]
Adults	SjIR	Insulin receptor enabling glucose uptake mediating parasite growth and reproduction	
?	SjMF	Myoferlin, a part of the ferrin family of tegument proteins involved in plasma membrane repair	[23]
All stages	SjASP	Aspartic protease responsible for digestion of haemoglobin	[18,24,25]
Adults	Sj Serpin	Serine protease inhibitor	[24,25]
Adult males	SjSVLBP	Very low density lipid binding protein	[18]
All stages	SmESP	Laval excretory and secretory products	[26]
?	SjNP30	Anti-idiotypic antibody	[27]
?	Ad.pIXgp70	Friend Virus (FV) surface envelope protein gp70	[28]
Egg stage	LNFIII	Lacto-*N*-fucopentaose III found on the surface of Schistosoma eggs	[29]

**Table 3:** Alum-adjuvanted *Schistosoma* vaccine candidates.

Adjuvant	Administration	Antigen ^a^	Reduction (%) ^b^		References
			Eggs	Worms	
Alum	s.c	Sm28GST	n.t	n.t	[30]
	?	Sm28GST	66 (primates)	38	[31]
	i.d	Sh28GST *(Bilhvax)*	n.t (primates)	66–77	[5,32,33]
	i.d	Sj97 pmy	33 (pigs)	n.t	[34]

^a^ Current vaccine candidates include Sj/SmTPI, Sj/Sm pmy, Sh28GST, Sm14, Sm-tsp-2, SjIR, Sj/Sm23 [3]; ^b^ Experimental data acquired in mice/rats unless specified; Abbreviations: n.t: not tested; i.d: intradermal; s.c: subcutaneous; Alum: aluminium hydroxide.

**Table 4:** Emulsion-adjuvanted *Schistosoma* vaccine candidates classified as a function of the adjuvant.

Adjuvant	Administration	Antigen ^a^	Reduction (%) ^b^		References
			Eggs	Worms	
RIBI (MPL-TDM)	s.c	Sm14	43–67	n.t	[32,35,36]
TiterMax	s.c	SmVAL4	n.t	n.t	[37]
	s.c	SmVAL26	n.t	n.t	[37]
	i.d	Sj97 pmy	34.5 (pigs)	n.t	[6,21,32,34]
FCA + IFA	i.d	Sm-tsp-1	34	52	[32,38]
	i.d	Sm-tsp-2	57	64	[32,38]
	s.c	Smteg	n.t	45	[39]
	s.c	Sm22,6	34	n.t	[18,32]
	s.c	Smteg	n.t	2–18	[39]
	i.p	Sm-CatD	n.t	n.t	[20,32]
	i.p	Sj Serpin	n.t	36	[18,40]
	s.c	Sjc26GST	23	59	[6,21,32,41]
	s.c	Sb28GST	37 (sheep)	18	[22,32]
	s.c	Fh15FABP	72	n.t	[32,42]
FCA	s.c	Pmy	n.t	40	[43]
	s.c	Sm28GST	n.t	n.t	[18,30,44]
	i.d	Sjc-97 pmy	34–39	n.t	[6,21,32]
	i.d	SjFABP	32	n.t	[6,21,32]
	i.m	SjFABP	59 (sheep)	23–70	[6,21,32]
	s.c	SjFABP	33	47	[6,21,32]
	i.d	SjFABP	49	n.t	[32]
	i.d	Sjc26GST	n.t (pigs)	53	[6,21,32]
	i.m	Sjc26GST	62 (sheep)	38	[6,21,32]
	i.m	Sj23	58–66 (sheep)	35–58	[6,21,32]
	s.c	Sjc26GST (SjGP-1)	27	27	[45]
	s.c	Sjc26GST(SjGP-2)	10	4.2	[45]
	s.c	Sjc26GST(SjGP-3)	15	37	[45]
	s.c	Sjc26GST(SjGP-4)	16	14	[45]
	?	Sj23	n.t	59 (sheep)	[21]
	s.c	Sb28GST	46 (goats)	35	[32]
	i.m	Sb28GST	50 (calves)	89	[32]
	i.n	Pmy	n.t	n.t	[43]
IFA	s.c	Sjc26GST(SjGP-3)	26	30	[45]
GLA-SE	?	Sm-p80	40–53 (primates)	25	[5,46]
	?	Sm-p80	n.t (hamster)	48	[5,46]
Montanide IMS 1312	s.c	Sjc26GST(SjGP-3)	35–37	17–23	[45]
Montanide ISA 70M	i.m	Sj62, Sj28, Sj23, Sj14-3-3	n.t	40	[24,25,47]
Montanide ISA 206	s.c	SjMF	28	23	[23]
	s.c	Sjc26GST(SjGP-3)	14	25	[45]

^a^ Current vaccine candidates include Sj/SmTPI, Sj/Sm pmy, Sh28GST, Sm14, Sm-tsp-2, SjIR, Sj/Sm23 [3]; ^b^ Experimental data acquired in mice/rats unless specified; Abbreviations: n.t: not tested; i.m: intramuscular; i.d: intradermal; s.c: subcutaneous; p.c: percutaneous; i.p: intraperitoneal; i.t: intratracheal instillation; FCA: Freund Complete Adjuvant; IFA: Incomplete Freund Adjuvant; GLA-SE: glucopyranosyl lipid adjuvant-stable emulsion; MPL-TDM: monophosphoryl lipid A + trehalose dicorynomycolate; R-848: resiquimod; Pmy: paramyosin.

**Table 5:** Particulate-adjuvanted *Schistosoma* vaccine candidates classified as a function of the adjuvant.

Adjuvant	Administration	Antigen ^a^	Reduction (%) ^b^		References
			Eggs	Worms	
QuilA	s.c	Sj97 pmy	32	66	[6,21,32]
	i.m	Sj97 pmy	34 (water buffalo)	48	[6,21,32]
	i.m	SjSVLBP	n.t	34	[18,48]
	i.m	Sj97	50–80	35–40	[24,25,49]
	s.c	SjASP	n.t	21–40	[3,18,50]
	i.p	SjLD2 SjIR	67	37–42	[5,51]
Liposome	i.t	SjGST1194	0	0	[52]
	orally	Sm28GST	53	52	[53]
PGN	s.c	SmESP	n.t	n.t	[26]
Vegetal polysaccharide	i.n	Pmy	n.t	40	[43]
Lewis X polysaccharide	i.n	LNFPIII	n.t	n.t	[29]
Poly(I:C)	s.c	SmESP	n.t	n.t	[26]
CpG ODN	?	Sm-p80	n.t (primates)	58	[54,55]
LCP	i.p	Sm-CatD	n.t	n.t	[20,32]
R-848	i.m	Sm-p80	100	70	[5,56]
	?	Sm-p80	n.t (primates)	52	[54]

^a^ Current vaccine candidates include Sj/SmTPI, Sj/Sm pmy, Sh28GST, Sm14, Sm-tsp-2, SjIR, Sj/Sm23 [3]; ^b^ Experimental data acquired in mice/rats unless specified; Abbreviations: n.t: not tested; i.m: intramuscular; s.c: subcutaneous; i.p: intraperitoneal; i.t: intratracheal instillation; LCP: Lipid core peptide; QuilA: saponin; R-848: resiquimod; Pmy: paramyosin; PGN: peptidoglycan; poly(I:C): polyinosinic:polycytidylic acid.

**Table 6:** Cytokine-adjuvanted *Schistosoma* vaccine candidates classified as a function of the adjuvant.

Adjuvant	Administration	Antigen ^a^	Reduction (%) ^b^		References
			Eggs	Worms	
IL-12 plasmid adjuvant	i.m	Sj23	22–28	27–35	[24,25]
	i.m	SjTPI	44–53	30–33	[24,25]
	i.m	SjTPI ^c^	13–60	21–53	[21,57]
	i.m	SjTPI (native)	47–53 (pig)	48–53	[21,57]
	?	SjTPI	18	32	[21]
	?	Sj23	48–59	30	[21]
	s.c	Sj PV1223	80	66	[58]
IL-4	s.c	Ad.pIXgp70	n.t	n.t	[28]
IL-18	i.m	Sj26GST	53.0–56.6	49.4	[59]
TSLP	s.c	SmESP	n.t	n.t	[26]
	s.c	rSG3PDH/PRX-MAP	33–66.9	69.3	[60]

^a^ Current vaccine candidates include Sj/SmTPI, Sj/Sm pmy, Sh28GST, Sm14, Sm-tsp-2, SjIR, Sj/Sm23 [3]; ^b^ Experimental data acquired in mice/rats unless specified; ^c^ Cocktail vaccination; Abbreviations: n.t: not tested; i.m: intramuscular; s.c: subcutaneous; TSLP: thymic stromal lymphoprotein; SG3PDH: glyceraldehyde 3-phosphate dehydrogenase; PRX-MAP: peroxiredoxin.

**Table 7:** Microbial-adjuvanted *Schistosoma* vaccine candidates classified as a function of the adjuvant.

Adjuvant	Administration	Antigen ^a^	Reduction (%) ^b^		References
			Eggs	Worms	
BCG	i.d	Pmy	33	n.t	[16,32]
	i.d	Sm97 pmy	n.t	n.t	[5,18,61]
Cholera Toxin	i.p	SjNP30	n.t	53	[27]

^a^ Current vaccine candidates include Sj/SmTPI, Sj/Sm pmy, Sh28GST, Sm14, Sm-tsp-2, SjIR, Sj/Sm23 [3]; ^b^ Experimental data acquired in mice/rats unless specified; Abbreviations: n.t: not tested; i.d: intradermal; i.p: intraperitoneal; BCG: *Bacillus* Calmette-Guérin; Pmy: paramyosin.

**Table 8:** Other-adjuvanted *Schistosoma* vaccine candidates as a function of the adjuvant.

Adjuvant	Administration	Antigen	Reduction (%) ^a^		References
			Eggs	Worms	
SmCB1 (papain)	s.c	-	26.6–51.3	60.0–66.1	[62]
	s.c	SG3PDH/PRX-MAP	54.6–58.4	75.0–83.7	[62]
FhCL1 (papain)	s.c	-	34.9–58.8	56.4–60.4	[62]
	s.c	SG3PDH/PRX-MAP	60.1–65.9	66.4–73.4	[62]

^a^ Experimental data acquired in mice/rats unless specified; Abbreviations: s.c: subcutaneous; SG3PDH: glyceraldehyde 3-phosphate dehydrogenase; PRX-MAP: peroxiredoxin.

The advantages of Alum as an adjuvant are the strong stimulation of antibody secretion, its proven clinical safety, the low production cost, and the ease of formulation and scale up [6]. However, it does also have significant limitations such as insufficient immunoprecipitation in comparison with other adjuvants, a low production of Th1-mediated and CTL cellular responses, and the potential risk of inducing allergic-type eosinophilic responses [6].

### 2.2. Emulsions

Emulsion-based adjuvants include two types, water-in-oil and oil-in-water. The oily phase is from the long carbon organic compound squalene, a natural component of cell membranes and precursor of cholesterol found in shark liver oil, although plant sources are now being utilised (rice bran, wheat germ, and olives). Oil adjuvants are known to induce a depot effect with sustained antigen release [63].

#### 2.2.1. RIBI

RIBI (containing monophosphoryl lipid A, MPL and trehalose dicorynomycolate, TDM) is an example of an oil-in-water emulsion adjuvant. The antigen is mixed with metabolisable oil (squalene) which is emulsified with saline containing Tween 80. RIBI also contains refined mycobacterial products, which act as an immunostimulant, and bacterial monophosphoryl lipid A (MPL). RIBI interacts with the membranes of immune cells resulting in cytokine induction and enhanced antigen uptake, processing and presentation, stimulating a non-toxic humoral and cell mediated immune response [64].

In 2001, Tendler and colleagues showed RIBI gave no enhancement of the protective immune response after vaccinating mice with the recombinant Sm14 protein against *Schistosoma mansoni* [35]. Since this study, preclinical research using RIBI as an adjuvant in helminth vaccines has been limited [32].

#### 2.2.2. TiterMax

TiterMax adjuvants, designed as an alternative to Freund’s Complete Adjuvant (FCA), are less toxic and contain no biological materials [63]. TiterMax adjuvant forms a microparticulate water-in-oil emulsion with a copolymer and metabolisable squalene oil [37] and is composed of mixtures of surfactant and linear, block or chains of non-ionic copolymers polyoxypropylene (POP) and polyoxyethylene (POE) [37,63]. The adjuvant-active copolymer forms hydrophilic surfaces which activate complement, immune cells and increases the expression of major histocompatibility molecules class II (MHC II) on macrophages, in addition to producing considerable levels of IgG1 and IgG2a antibodies [37]. TiterMax Gold is an improved version of the traditional TiterMax adjuvant [37].

Preclinical research on a TiterMax adjuvanted *S.* *mansoni* Venom Allergen Like (SmVAL) protein by Leite *et al.* [19] showed a significant production of IgG2a antibodies. SmVAL antigens, in addition to playing an important role in the host-pathogen interaction, are also involved in the recruitment of mast cells and basophils, inducing secretion of histamine and facilitating parasite invasion through vasodilation of skin [19]. This study focused on identifying and characterising allergic characteristics of this class of molecule, an important aspect in vaccine development. Here, the levels of specific IgG1 and IgG2a and the IgG1/IgG2a ratio indicated that immunisation with TiterMax Gold induced a more balanced (Th1/Th2) response, but the IgE serum levels revealed significantly higher levels only in the rSmVAL4 vaccinated group when compared with all other groups in the study (PBS/Alum control, rSmVAL26 and rSmVAL4-Pro) [37]. In this study, TiterMax Gold was chosen as it is a less Th2 prone and more balanced adjuvant. This promotion of IgE responses poses a problem in vaccine design as it may elicit undesirable side effects, including allergic responses. Further analysis of TiterMax as an adjuvant in vaccines for schistosomiasis requires evaluation of the levels of IgE-specific antibodies for specific proteins in sera, in addition to the screening and mapping of IgE epitopes on potential vaccine candidates [37].

TiterMax has been sparingly used to adjuvant helminth vaccines [32] and a recent study comparing TiterMax Gold with QuilA and FCA on recombinant antigens of the liverfluke (trematode) parasite *Fasciola hepatica* indicated TiterMax demonstrated no increased protective effect in terms of the reduction of fecal egg count, relative to the negative control PBS (Table 4) [37]. This study also observed an adjuvant effect on weight gain of vaccinated animals with TiterMax Gold giving significantly higher weight gain relative to the other adjuvants tested. Together, these results demonstrated that the mechanism of action against parasitic disease is not always the same as the mechanism of protection against challenge infection or parasite growth and development, and that, ultimately, the development of targeted adjuvants specifically designed for individual vaccines is a promising technology [32,37].

#### 2.2.3. Freund’s Complete Adjuvant

FCA is a water-in-oil emulsion that localises antigen for slow release within the immunised host for up to six months. It contains mineral oil, the surfactant mannide monoleate and heat killed *Mycobacterium tuberculosis*, *Mycobacterium butyricum* or their extracts (for aggregation of macrophages at the inoculation site) [21]. FCA promotes both cell mediated and humoral immunity primarily via toll-like receptor (TLR) activation but, in some instances, this has also been shown to induce excessive inflammation at the injection site [63]. A further disadvantage of this adjuvant is that it can produce some immunological toxic effects due to the non-metabolisable mineral oil and for this reason, it is only registered for use on laboratory animals.

Currently, FCA is the most common adjuvant used in the preclinical evaluation of schistosome vaccine candidates (Table 4) [32]. Preclinical research on FCA adjuvanted *Schistosoma* vaccines shows the induction of a mixed Th1/Th2 immune response with minimal evidence of toxicity or allergic reactions [19,38].

Immunisation of mice with *S.* *mansoni* tegument (Smteg) antigen, in combination with Freund’s adjuvant, reduced parasite burden (43%–48%), egg production and disease morbidity, generating an immune response characterised by IFN-γ, IgG1, and IgG2c production leading to parasite tegument damage *in vivo* (Table 4) [39]. In the absence of adjuvant, a significant production of antibodies occurred but these failed to induce a protective immune response [22]. Additionally, no reduction in worm burden, egg numbers in the liver and intestine, nor female fecundity was observed compared with the control (saline) group [39]. Additionally, a lack of protection was associated with IL-10 production and impaired antigen presentation to CD4^+^ cells. In this instance, immunisation of mice with Smteg stimulated the production of molecules involved in antigen presentation and Th1 polarisation. However, in the absence of an adjuvant to promote an immune response and to enable prolonged antigen release in the host thereby improving uptake, the rapid clearance of Smteg from the injection site explains the weak immune response generated and the absence of protective immunity [39].

Serpin proteinase inhibitors (serpins) are an important superfamily of endogenous inhibitors that regulate proteolytic events active in a variety of physiological functions [40]. Immunisation of rabbits with tegument localised *S. japonicum* serpin, in combination with FCA and Incomplete Freund’s adjuvant (IFA) induced the production of high levels of IgE and IgG1 subclass antibodies, a marked IL-4 response, and B cell (CD19) proliferation. These all contributed to a predominant Th2-type response [40]. Further, this type of immune response was also confirmed when mice immunised with this antigen/adjuvant system developed moderate protection against *S. japonicum* infection resulting in a 36% and 39% reduction in adult worms and eggs, respectively (Table 4) [40].

The family of glutathione-*S*-transferase (GST) enzymes is present in all organisms and it plays an important role in the detoxification of electrophilic compounds. In 1995, McManus *et al*. [41], vaccinated mice using the 26 kDa purified recombinant *S. japonicum* GST (reSjc26GST) antigen in combination with FCA (with IFA boost) to determine the effect on worm burden and fecundity. This study found a moderate but significant reduction in worm numbers (23.7% and 26.4%) when compared with the controls ((adjuvant only; FCA and IFA in PBS) and challenge (PBS only), respectively [41]. There was also a significant decrease in the number of liver and spleen eggs in the challenge group compared with the challenge control, but an unexplained significant increase in eggs (both in the spleen and liver) in the adjuvant control group [41]. Further immunisation studies have been performed by McManus and colleagues [45] using chimeric proteins formed from Sjc26GST conjugated to paramyosin fragments (namely SjGP-1 to SjGP-4). Here, adjuvant selection was evaluated on the immunogenicity and protection efficacy of chimeric proteins in mice. Emulsion-based adjuvants compared in this study included FCA and montanides ISA 206, IMS 1313, and ISA 70M [45]. The results revealed that SjGP-3 formulated with FCA generated a moderate mixed response (Th1/Th2) after immunisation, which was consistent with montanide ISA 206. However, no significant reduction in liver egg or worm burden was observed compared with the experimental control (PBS) group when FCA was used as adjuvant (Table 4) [45]. These studies demonstrated a relatively low but significant protection (in terms of reduced worm numbers) was obtained in the challenge groups with the level of antibody higher in immunised mice compared with the controls [41,45].

Further, in a murine challenge model using FCA (and IFA boost) adjuvanted *S. mansoni* tetraspanins (Sm-tsp-1 and Sm-tsp-2), a significant reduction in worm and egg burdens was obtained for both antigens (Table 4), when compared with a PBS control [38]. Additionally, the antibody response was dominated by IgG1 and IgG2a antibodies, consistent with a study by Oliveira *et al.* in 2005 [19]. In this latter study, immunisation with a *S.* *mansoni* lung-stage rSm22.6 antigen in the presence of FCA resulted in a mixed Th1/Th2 type immune response (IgG1/IgG2a profile) compared with the control group (FCA in PBS) [19]. This combined humoral and cellular response is thought to be the key in generating maximal immunity to schistosomes [19]. A clinical trial with the Sm-tsp-2 vaccine is due to take place following toxicology studies [65].

#### 2.2.4. Incomplete Freund’s Adjuvant 

IFA has the same formulation as FCA but does not contain mycobacterium. IFA was designed to minimise the excessive inflammatory effects observed with FCA [64] and, subsequently, its use has been generally limited to booster doses of antigen as it is less effective than FCA for primary antibody induction. Consequently, preclinical research using IFA-adjuvanted *Schistosoma* vaccines is in conjunction with FCA, where IFA is used to boost the initial FCA immunisation. The low ability of IFA to produce antibody is supported by research by Arakawa and colleagues in 2010 which showed that subcutaneous immunisation of *S. japonicum* 97 kDa myofibrillar protein paramyosin emulsified with cholera toxin was more potent that emulsification with IFA [43].

#### 2.2.5. Glucopyranosyl Lipid Adjuvant-Stable Emulsion

Glucopyranosyl lipid adjuvant-stable emulsion (GLA-SE) is a two-part adjuvant system containing GLA, a formulated synthetic toll-like receptor 4 agonist (promotes a Th1 response), and a SE of oil-in-water (promotes a Th2 response) [66,67]. As an adjuvant, GLA-SE has been used in Phase I clinical trials with flu (Fluzone and PanBlock) and TB (ID93) vaccines [66,67]. These studies have shown GLA-SE adjuvanted flu vaccines increased humoral and cellular immunogenicity, characterized by a Th1 biased immune response and caused no adverse side effects in animals [67]. Additionally, flu vaccines adjuvanted by GLA-SE had enhanced potency in terms of antibody and T cell responses, and a broadened serological response compared with the flu vaccine alone [38]. Further, in studies using TB as the vaccine antigen, both SE and GLA-SE induced potent cellular responses in a murine model. GLA-SE induced multifunctional CD4^+^ Th1 cell responses (IFN-γ, TFN-α, and IL-2) along with significant protection in mice and guinea pigs. No protection was observed with SE alone [67].

Preclinical research on the GLA-SE adjuvanted *S. mansoni* Sm-p80 vaccine resulted in a pronounced reduction in worm burden (48%) in hamsters. Similarly, vaccination of *S. haematobium* with Sm-p80 gave a reduction in worms (25%) and egg (64%) burden in primates (Table 4) where appropriately balanced pro-inflammatory (Th17 and Th1) and Th2 biased immune responses were induced, appearing to be essential for protection against schistosome challenge [46]. Notably, cross-species protection was also observed, coupled with prophylactic, therapeutic and anti-fecundity efficacy against *S. mansoni* homologous parasites. Further preclinical development leading to human trials is currently underway reinforcing GLA-SE as a promising *Schistosoma* adjuvant [46]. To date, however, clinical trials using GLA-SE adjuvanted *Schistosoma* vaccines have not been undertaken.

#### 2.2.6. Montanide

Montanides ISA 206, IMS 1312 and ISA 70M are water-in-oil microemulsions composed of squalene stabilised with surfactants [45]. More specifically, ISA 70M is a water-in-oil emulsion, ISA 206 is a water-in-oil-in-water emulsion, and IMS 1312 is an emulsion nanoparticle [45]. Montanides produce strong antibody secretion, T cell proliferation, and a balanced Th1/Th2 cytokine profile. Montanides are also known to produce a depot effect, recruit, activate and induce the migration of APCs to lymph nodes, and moreover, they interact with cellular membranes favouring antigen uptake [6].

Several Montanide adjuvanting vaccines against *Schistosoma* have been or are currently in preclinical evaluation. Pan *et al.*, investigated the adjuvanting effect on the protection of the Sj26GST (SjGP-3) antigen [45]. The results revealed that SjGP-3 formulated in veterinary adjuvant ISA 70M induced a lasting polarised Th1 immune response, whereas the other adjuvants in the study (FCA, ISA 206 and IMS 1312), generated a moderate mixed Th1/Th2 response after immunisation with all except IMS 1312 shifting to a Th2 response after the onset of egg production. More importantly, the SjGP-3 ISA 70M adjuvanted formulation induced significant reductions in liver egg burden (47%–50%) and the number of liver eggs per female worm (34%–37%), but there was less effect on worm burdens, reduced by 17.3%–23.1%. No effect on the number of liver eggs per female was observed with the other adjuvants [45]. The same antigen formulated with FCA or ISA 206 induced a moderate mixed Th1/Th2 immune response post vaccination, but this mixed immune response shifted to a Th2 response after parasite maturation and the onset of egg production [45]. This mixed immune response was also observed by Lin *et al.*, who used ISA 206 as an adjuvant for the SjMF antigen in a murine model [23]. This study found reduced worm burdens (21.8%–23.2%) and liver egg number (42.5%–28.3%) [23]. Notably, the novel adjuvant ISA 70M provided an enhanced humoral and cell-mediated immune response in this study; it comprises a high grade injectable mineral oil and an extremely refined emulsifier obtained from mannitol and purified oleic acid of vegetable origin, and is a modified version of the successful veterinary adjuvant, ISA 70. Hence, there is promise for this adjuvant for applications in humans [45].

The advantage of Montanides is their capacity to stimulate both humoral and cellular responses. However, Montanide use in malaria vaccines has been associated with side effects including pain and inflammation at the injection site reported in several studies. These concerns, in addition to the stability of malaria antigens in Montanide adjuvanted vaccines and the cost of production for each antigen, raises potential issues with further experiments required to ensure the safety and feasibility of Montanide use in *Schistosoma* vaccines [6]. To date, no clinical trial with an adjuvanted Montanide *Schistosoma* vaccine has been undertaken.

### 2.3. Particulate

#### 2.3.1. Saponins

The adjuvant properties of saponins were first demonstrated in the early 1920s, and in 1950 saponin, was used in a veterinary vaccine against foot and mouth disease [68]. Saponins are natural glycosides of steroids or triterpenes widely distributed in plants and animals. QuilA, an extract of *Quillaja sapomari*, and its derivatives constitute the most extensively used saponins for adjuvant purposes. QuilA is not suitable for human use as it is a heterogeneous mixture; however, isolation and analysis of individual fractions (e.g., QS21) from QuilA as adjuvants has been initiated. Saponin-type adjuvants have been shown to stimulate specific humoral and cellular immune responses including generation of Th1 cytokines, cytophillic antibodies and strong antigen-specific CTL responses [63]. Although the mechanism of action of QuilA is currently not well understood, it has been shown to have a low level of toxicity [15].

Saponins, isolated from *Atriplex nummularia*, were found to be suitable immunostimulatory adjuvants in a study investigating the cross-reactivity between *S. mansoni* and *Fasciola gigantica* adult worms and egg homogenates of the parasites by looking at IgM titres [69]. Additional studies using a combination of QuilA and the recombined protein SjLD2 (ligand domain of *S. japonicum* insulin receptor 2) showed a reduction in fecal eggs, stunting of adult worms and a reduction in liver granuloma density compared to the control (QuilA in PBS), with potential use as a transmission blocking vaccine [51]. Further, protein fragments of paramyosin formulated in QuilA were found to be highly immunogenic in mice and showed promising protective efficacy in terms of significant reductions in worm and egg numbers, but there was no apparent correlation between the antibody titres generated and the level of protective efficacy obtained [49].

#### 2.3.2. Liposomes

Liposomes are lipid bilayer vehicles made up of lipid membranes containing phospholipids and other lipids in a bilayer configuration separated by aqueous compartments. Cationic liposome formulations are lipid bilayer vesicles with an overall positive surface charge; however, in some cases cationic liposomes have been shown to be insufficiently immunostimulatory and are often combined with other immunostimulatory factors. Liposomes are considered safe for vaccine use [70].

A *S. mansoni* GST vaccine study was performed using negatively charged cationic liposomes in a murine model. Results showed high IgG2a/IgG1 and IgG2b/IgG isotype ratios and IFNγ/IL-4 cytokines. This implied that immunisation with liposome-mediated conformational variants of the *S. mansoni* hybrid immunogen GST1194 is beneficial to the development of an immune response towards a Th1 phenotype [52]. However, a reduction in the number of female worms was not obtained when the antigen was given alone, suggesting that the observed impact on female development is related to the generation of a strong cellular and humoral Th1 immune response [52].

In an earlier study, mucosal immunisation with Sm 28 kDa glutathione S-transferase using liposomes generated both mucosal and systemic immune responses including IgA production in the gut and IgGl/IgG2b antibodies in the sera. This study also showed the antigen was present on both the inner and outer membranes of the liposome vesicles and that the main antigenic features of the antigen were conserved [53]. These results open further doors for the use of liposomes in mucosal *Schistosoma* vaccines, especially in the clinical context.

#### 2.3.3. Polysaccharides and Oligosaccharides

##### 2.3.3.1. Peptidoglycan

Peptidoglycan (PGN, also known as Murein), a component predominately found in the cell wall of Gram-positive bacteria, is reported to interact with TLR2/6 heterodimers on lymphoid and non-lymphoid cells, inducing prominent Th2-biased immune responses and a low level of Th1 cytokines [26]. The basic structure of PGN has a carbohydrate backbone of alternating units of *N*-acetylglucosamine and *N*-acetylmuramic acid, with the *N*-acetylmuramic acid residues cross-linked to a short chain peptide. Synthetic versions of PGN and other PGN-component derivatives have been extensively studied as potential adjuvants for human use, but significant side effects such as fever and inflammation at the site of injection have prevented their acceptance for use in clinical trials [71]. Applications of PGN as an adjuvant in *Schistosoma* research has been outlined under *Synthetic Polynucleotides* in Section 2.3.5.

##### 2.3.3.2. Plant-Based Polysaccharides

The vegetal polysaccharide (a conjugation of tea and mushroom polysaccharides) used by Youen *et al.* [58], and the fungal polysaccharide Lentinan, a known T cell adjuvant, used by Lichtenberg *et al.* [72], are examples of plant-based adjuvants used in schistosome vaccines (Table 5). Although not common, these adjuvants reduced schistosome fecal egg counts [58,72] and the vegetal adjuvant study, also resulted in reduced worm burden (66%), but no significant difference in IgG titre was observed post-challenge. These studies show that plant-based polysaccharides are able to promote the efficacy of a vaccine, but additional studies are required to determine their direct modes of action [58].

##### 2.3.3.3. Lewis X Polysaccharides

A number of glycan-based assemblies including Lewis X tetrasaccharides have been identified and isolated from helminths. Lewis X, originally identified from the egg stage of *S. mansoni,* has now been shown to be common among many helminths. Lewis X and its derivatives are known to drive Th2-biasing and immunomodulatory properties *in vivo* and *in vitro* without the need for an additional adjuvant [9]. A review by Harn *et al.* (and references within) describes, in detail, the immunodulatory function of Lewis X derivatives as a potent adjuvant [9].

A Lewis-type carbohydrate-Lacto-*N*-fucopentose III (LNFPIII) that contains the Lewis X trisaccharide was isolated from *S. mansoni* egg antigens and has recently been demonstrated to be a potent inducer of a Th2-type response. Alone, synthetic LNFPIII produces a small immunological effect associated with a Th2 response (B cell proliferation and IL-10) [9]. LNFPIII can also act as an adjuvant by inducing antibodies against coupled protein antigen (human serum albumin). This molecule may prove to be a useful adjuvant in the development of schistosome vaccines [24,29]. Additionally, the proliferative and IL-10 response to LNFPIII was assessed in PBMC from humans infected with *S. mansoni*. The results indicated that LNFPIII is a potent immunoreactive epitope, negatively influencing the response of PBMCs against parasite antigens via IL-10 production. However, a slight increase in IgE antibodies was also evident [73].

Interestingly, fucosylated sugars induce production of antibodies during *Schistosoma* infection, supporting the concept that carbohydrates are important for driving a Th2-biased response in all helminths. Other studies also conclude that Lewis X/LFNPIII can function as an adjuvant for unrelated, third-party antigens enhancing the Th2-type antibody response [9]. The use of small oligosaccharides as adjuvants and/or Th2 drivers is still at an early stage, but the findings of these studies clearly show that Lewis X and its derivatives possess novel and potent adjuvant qualities. Further, subsequent analysis on other *Schistosoma* glycans as potential adjuvants is required and this may provide information on disease states and responses [9].

#### 2.3.4. Synthetic Nucleotides

##### 2.3.4.1. Poly(I:C)

Polyinosinic:polycytidylic acid, commonly referred to as poly(I:C), is a synthetic double-stranded RNA adjuvant that is structurally similar to double-stranded RNA present in some viruses with one strand being the inosinic acid polymer, and the other strand being the cytidylic acid polymer [74]. It has been shown to activate an immune response through two distinct pattern recognition receptors, promoting Th1 biased immunity through the induction of IL-12 and type I IFNs [75]. Endosomal poly(I:C) naturally activates TLR3 (located in the endosomal compartment of specialised cells) inducing IL-12 and type I IFNs (IFN-α, IFN-β, and IFN-γ) production, and cytosolic poly(I:C) binds to cytoplasmic RNA helicase MDA-5, stimulating type I IFN. Poly(I:C) has also been shown to maintain long-lasting T cell immunity [74,75].

Poly(I:C) as a *Schistosoma* adjuvant has had limited applications (Table 5) with less than desired results. In a study by Tallima and colleagues in 2012 [26], poly(I:C) failed to induce production of IL-4 and subsequently led to elevated IFN-γ levels, signifying a Th1 response. In this study, IL-4 in mice was shown to be essential in *Schistosoma* resistance while susceptibility in rats was associated with the production of IFN-γ [26]. Notably, poly(I:C) is known to stimulate the production of key factors associated with the polarisation of dendritic cells towards a Th2 cytokine response. This is important as *Schistosoma* resistant or highly susceptible subjects were shown to be Th2 or Th2/Th0 positive [26]. Subsequently, in the study by Tallima *et al.*, a comparison between the adjuvants poly(I:C), PGN and thymic stromal lymphoprotein (TSLP) found PGN triggered heightened levels of IL-4, IL-17, and IFDN-γ, and TSLP succeeded in directing the immune response towards a Th2-biased profile [26]; however, negligible antibodies were generated. This study demonstrates how important it is to determine the type of immune response the candidate vaccine antigen induces in the host during natural infection, without the inclusion of an adjuvant, and how the type of protective immune response (Th1 or Th2, or a mixture) should be defined. Following this, adjuvant selection capable of augmenting or skewing the candidate vaccine antigen-induced immune response towards the desired Th-biased profile can be made [26]. No further studies using Poly(I:C) adjuvanted helminth vaccines have been reported.

##### 2.3.4.2. CpG-Motif Oligodeoxynucleotides

CpG-motif oligodeoxynucleotides (CpG ODNs) are short single-stranded synthetic DNA molecules that contain a cytosine triphosphate deoxynucleotide (“C”) followed by a guanine triphosphate deoxynucleotide (“G”). The “p” refers to the phosphodiester link between consecutive nucleotides, although some ODN have a modified phosphorothioate backbone instead. When these CpG motifs are unmethylated, they act as immunostimulants [76]. Although identified in 1893 as part of a mixture of bacterial cell lysate [77], it was not until 1995 when Klinman *et al.*, identified the CpG motif within bacterial DNA to be responsible for immunostimulatory properties, and since then, synthetic CpG ODNs have been the focus of intense research, including as vaccine adjuvants [78,79]. Additionally, Klinman *et al.*, were the first to describe Class B ODNs which are strong stimulators of human B cells and monocyte maturation and, for this reason, they have been studied extensively as therapeutic agents because of their ability to induce a strong humoral immune response making them ideal as vaccine adjuvants [78].

Reports of CpG ODN adjuvanted schistosome (Table 5) and other helminth [18] vaccines are limited, but a study immunising mice with Sm-p80-pcDNA3 antigen using CpG ODN as an adjuvant found a substantial increase in Sm-p80-mediated protection compared with the control group [55]. This result, in addition to the downregulation of cytokines important for B cell proliferation and the recovery of a higher number of parasites in antibody knockout mice, indicated that antibodies play a significant role in Sm-p80 protection [55]. However, the role of the adjuvant in this study could not be effectively determined since there was no adjuvant-only control. CpG ODN has also been used as an adjuvant for *S. japonicum* 26 GST DNA vaccine (pVAX1-Sj26GST) [80]. Vaccination with pVAX1-Sj26GST combined with CpG inhibited Treg immunosuppressive function, increased the responses of IFN-γ, tumor necrosis factor (TNF)-α, IL-4, 10, 2 and IL-6 and decreased Foxp3 expression *in vitro*, allowing expansion of antigen-specific T cells against pathogens. Using TLR7/8 (R848) and TLR9 (CpG ODN) as potent adjuvants for pVAX1-Sj26GST vaccination also induced a similar immune response against *S.japonicum* challenge [81].

While the main issue for TLRs is that the combination of antigen and TLRs diffuse rapidly after injection, so if they are encapsulated into nanoparticles, then they will stay by the draining lymph node and boost the response tremendously as demonstrated in Kasturi *et al.*’s study [82]. The immunization of mice with synthetic nanoparticles containing antigens plus TLR4 and 7 can induce synergistic increases in antigen-specific, neutralizing antibodies compared to immunization with a single TLR ligand. Additionally, the formulation and administration protocol of vaccines is also important. For example, a prime/boost strategy for the administration of DNA-based vaccines has been shown to enhance protection, and decrease worm and egg burden, as DNA vaccination is generally less effective than protein [56,83,84,85,86,87].

#### 2.3.5. Peptide Analogues

Self-adjuvanting lipopeptide vaccines offer an alternative to traditional adjuvant systems and are an easier platform for large-scale production [13]. They consist of a synthetic, non-microbial, lipopeptide adjuvant based on lipo-amino acids attached to numerous polylysine branches [12,20]. These branches provide a scaffold for conjugation of multiple peptide epitopes. The synthetic lipopeptide system has been shown to have immunostimulatory properties by targeting dendritic cells [20]. This system has undergone rigorous pre-clinical assessment in murine vaccine trials for Group A *Streptococcus*, cancer and malaria [20]. Additionally, long lasting high titre antibody responses have been reported when using lipid core peptides (LCPs) constructed with the conserved epitopes of the *Streptococcus pyrgens* M protein and these have been shown to induce protective immunity in a murine disease model [20].

Preclinical research using lipid-adjuvanted *Schistosoma* vaccines has recently been investigated utilising the *S. mansoni* cathepsin D hemoglobinase protein [20]. Mice immunised with LCP adjuvanted peptides induced stronger IgG1 responses compared with the FCA adjuvant. No IgG2 antibody was generated in LCP or FCA adjuvanted systems. However, inconsistent antibody responses were found in separate mice experiments necessitating further studies, including the addition of a universal T helper epitope to boost IgG titres and subsequently the humoral response [20]. Further investigation into the development of self-adjuvanting peptide antigens containing multiple epitopes offers great potential for schistosome vaccines.

#### 2.3.6. Imidazoquinolines

Imidazoquinoline compounds are double cyclic synthetic molecules including resiquimod (R-848) which are TLR7/8 ligands [6]. These compounds have been shown to improve both antibody and T cell responses following administration using diverse routes. Additionally, R-848 has been approved for topical treatment of malignant and non-malignant disorders [6]. With regard to *Schistosoma*, intramuscular administration with Sm-p80 in a murine model gave significant reductions in liver/intestinal eggs (100% in mice/primates) and worms (70% in mice and 60% in primates) [5,56]. Additionally, it was shown that R-848 slightly boosted the protective effects of Sm-p80 as correlated by antibody production. Here, IFN-γ and IL-2 was produced indicating that the immune responses were Th1 biased [56]. In human correlate studies, Sm-p80 specific IgG reactivity with human sera has been shown. However, low IgE was lacking in the prevailing high-risk population, minimising the risk of hypersensitivity. From these data, Phase I and II clinical trials have been proposed with the aim of using Sm-p80 as a blocking vaccine [88].

Subsequently, R-848, and the TLR9 ligand, CpG ODN, as adjuvants for pVAX1-Sj26GST have been assessed. Here, antigen in combination with R-848 and CpG ODN increased splenocyte proliferation and IgG/IgG2a levels. However, a decrease in CD4^+^/CD25^+^ and regulatory T cells frequency was observed *in vivo*. This is indicative of enhanced protection against *S.* *japonicum* as both adjuvants inhibited T regulatory mediated immunosuppression and up regulated the production of interferons (TNF-α, IL-4, IL-10, IL-2, and IL-6) [81]. These factors have been shown to contribute to the expansion of antigen-specific T cells against pathogens. Overall, TLR ligand R-828 shows promising protective efficacy against schistosomes [81].

### 2.4. Cytokines

Several cytokines are under evaluation as vaccine adjuvants, including; IL-2, IFN-γ, granulocyte-macrophage colony stimulating factor (GM-CSF), TSLP, and more recently IL-12 [14]. Cytokines, as natural proteins intimately involved in the normal immune response, have great appeal as vaccine adjuvants. They are secreted by a variety of cells and are known to mediate the adjuvant effect of biological products including lipopolysaccharides, and until recently, have only been considered as possible immune adjuvants. Advantages of cytokines as adjuvants include limited allergic response, since they are natural proteins, and they have been shown to be effective in a variety of animal vaccine models including viral, bacterial and parasitic [89].

There are a number of ILs used in vaccine formulations and they all act on the immune system in a variety of different ways (reviewed by Rubin *et al.*, and references within) [79,89]. Of these, IL-4, IL-12 and IL-18 have been investigated in preclinical *Schistosoma* vaccine studies (Table 6).

#### 2.4.1. IL-12, IL-4, IL-18

IL-12 is an exceedingly potent adjuvant promoting IFN-γ release by IL-12 receptor-expressing T and natural killer cells inducing Th1 polarisation, as well as proliferation of IFN-γ T cells [90]. IL-12 is thought to play a pivotal role in the immunomodulatory activities of various immunologic adjuvants [14]. In a study by Wilson and colleagues [91], recombinant IL-12 was administered to mice in conjunction with an antigen derived from the lung-stage larvae of *S. mansoni* and elicited a dominant population of antigen-specific Thl cells, identified by increased levels of IgG2a and decreased levels of IgG1 and IgE. In contrast, antigen alone induced a mixed population of Thl and Th2 cells with secretion of IFN-y, IL-4, and IL-10. This study concluded that IL-12 was sufficient to elicit moderate but highly specific levels of protective immunity against challenge infection [91]. Th2 suppression, a reduction in worm burden and an increase in *S. mansoni* protection by IL-12 was also confirmed by Sher *et al.* [92]. Additionally, the most effective DNA vaccine on the market to date, Sm-p80 + IL-12 (calpain), has shown a 57% rate of protection in mice using IL-12 as an adjuvant [93]. Additionally, it has been reported that IL-12 was used as an adjuvant in SjC23 (*S. japonicum* tetraspanin 23 kDa integral membrane protein) and SjTPI (*S. japonicum* triose-phosphate isomerase) DNA vaccine trials in water buffaloes [94] and SjTPI DNA vaccine trails and pigs [95]. Here, IL-12 drives the immune response towards a Th1 direction, enhancing the protective immune effect of the vaccine [96].

IL-4 is an essential cytokine for the development of Th2 CD4^+^ cells from Th0 CD4^+^ cells, which drives Th2 development, and at the same time suppresses Th1 development. Additionally, IL-4, in combination with other cytokines, mediates proliferation and maturation of B cells in plasma, aiding antibody production [28,97]. Sadek *et al.* [98] evaluated IL-4 and IL-12 adjuvanted *S. mansoni* tegumental antigens and found that the use of IL-12 as an adjuvant significantly reduced the worm burden and liver egg count compared with the corresponding controls. In addition, histopathological examination of liver sections in the IL-12 tested groups showed a decrease in the size and number of granulomas and liver cell apoptosis. On the other hand, the results of the tested groups that received IL-4 as an adjuvant were contradictory and the authors concluded that IL-12 potentiated the protective effect of the *S. mansoni* tegumental antigen vaccine and appeared to be a useful adjuvant, while IL-4 was less effective [98].

The more recently discovered IL-18 has both biological and synergistic properties similar to IL-12 and has been shown to bias a Th1 type immune response [99]. This was confirmed in a study by Lin *et al.* [59] who vaccinated mice with a *S. japonicum* recombinant plasmid, pVAX/mIL-18, containing murine IL-18. Post vaccination, a significant increase in the production of IFN-γ and IL-12 was observed indicating that IL-18 enhanced the Th1-dominant immune response. Additionally, a reduction in worm (49.4%) and egg (50.6% hepatic and 56.6% fecal) burden was also noted (Table 6) [59].

There are numerous reports of Phase II clinical trials for applications in the treatment of cancer using IL-12 as an adjuvant. Notably, induction of peptide-specific CD8^+^ T cells and low toxicity has been recorded [90]. Additionally, a drawback of cytokine studies, as detailed in a minreview by Taylor in 1995 [100], describes how administration of cytokines can cause an imbalance of other cytokines present, influencing different antigen-specific and polyclonal responses. Ultimately, it is important for researchers to examine both antigen-specific and polyclonal responses when using cytokine adjuvants, especially considering a balance in homeostasis of the immune system is essential for immune responsiveness [100]. Additionally, the relatively short half-life of recombinant cytokines means they have limited applications as vaccine adjuvants unless conjugation to the antigen, or administration with liposomes or microspheres is developed for human-based applications [89].

Although significant interest using cytokines as adjuvants for *Schistosoma* vaccines has been described, to date, no clinical trials with helminth vaccines have been reported.

#### 2.4.2. Thymic Stromal Lymphoprotein (TSLP)

TSLP is a protein belonging to the cytokine family and plays an important role in the maturation of T cell populations through the activation of APCs. TSLP is an IL-7 short-chain hematopoietic cytokine that was initially cloned into mice as a B cell growth and differentiation factor, but in humans, it has been shown to mostly act on dendritic and mast cells [101]. The direct effect of TSLP on T cells remains controversial and, to date, no B cell effect has been reported [102]. As TSLP is a pro-allergic cytokine, extrapolation of vaccine trials from mouse to humans needs caution because of species-specific differences, and additionally, the potential toxicity of intranasal injection of TSLP needs to be considered [102].

Applications of TSLP as an adjuvant which are limited in *Schistosoma* research (Table 6) have been outlined in Section 2.3.5 (*Synthetic Polynucleotides*). Briefly, TSLP was successful in directing the immune response towards a Th2-biased profile, which was confirmed by appropriate cytokine production [26]. Additionally, studies by Tallima *et al*., analysed the adjuvanting effects of Th2-biased adjuvants TSLP and papain in *S. mansoni* and found significant reductions in worm burden and egg numbers compared to the unimmunised control mice post challenge (Table 6 and Table 8) [60].

### 2.5. Microbial Adjuvants

Bacterial toxins with adjuvant activity, such as Bacillus de Calmette et Guérin (BCG) and Cholera Toxin (CT), preferentially drive Th2-like responses and have been shown to enhance IgA and IgE antibody production. Adjuvants that drive Th2-like-immune responses could enhance protection against mucosal virus transmission by augmenting IgA protection [14,43].

#### 2.5.1. Bacillus Calmette-Guérin 

BCG (historically known as Vaccin Bilié de Calmette et Guérin) is a vaccine against tuberculosis [103,104,105]. BCG as an adjuvant is known to promote infection resistance associated with the stimulation of schistosome specific T cell dependent cell-mediated immune responsiveness, and the production of lymphocytes (IFN-γ) that activate macrophages for larval schistosome killing [61]. BCG is not commonly used in helminth vaccine development [32]. When BCG was used in combination with the schistosome antigen paramyosin, activation of macrophages leading to a reduction in schistosomula was observed and this was associated with the stimulation of T lymphocytes to produce lymphokines (e.g., IFN-γ). No protection was observed (Table 7) [61]. Interestingly, intraperitoneal immunisation was less effective than intramuscular vaccination which elicited protective immunity and the strain and source of BCG influenced the level of resistance [106]. Additionally, this protective immunity could be extended to other adjuvants including *Bordetella pertussis*, *Corynebacterium parvum* and saponin, but no protective immunity was observed with Alum [106]. Minimal humoral reactivity against all tested adjuvants was shown indicating protection in this model is based on cell-mediated immune effector mechanisms [106].

#### 2.5.2. Cholera Toxin

CT is commonly used for mucosal vaccine delivery [107], but a major drawback is its intrinsic toxicity [32]. CT has been evaluated in combination with schistosome vaccine candidates. Immunisation with *S. japonicum* paramyosin elicited no reduction in worm burden or fecundity, and no protection against parasite infection was observed despite an increase in serum antibodies responses [43].

Recent studies documenting the use of microbials as adjuvants in schistosome vaccine research is scarce indicating limited progress towards clinical stage vaccine trials with any CT or BCG-based vaccines (Table 7).

### 2.6. Protease

#### Papain

Schistosomes are known to express several different classes of cysteine peptidase proteins (e.g., the cathepsin family) which have major roles in the digestion of haemoglobin, reproduction, skin penetration, and more recently, biogenesis [108]. More specifically, cysteine peptidases isolated from papaya (from the papain superfamily) have been shown to skew immune responses towards a Th2 phenotype indicating their potential use as vaccine adjuvants [63,108]. In studies by Dalton *et al.* [63,108], papain was shown to have self-adjuvanting properties inducing significant protection (Table 8) against an experimental challenge using active papain infection with *S. mansoni* in a murine model [60]. Here, cysteine peptidases combined with *S. mansoni* vaccine candidates, glyceraldehyde 3-phosphate dehydrogenase (SG3PDH) and peroxiredoxin (PRX-MAP), conferred highly significant protection (up to 73%) against worm infection in an experimental challenge [62]. Increased protection and an up to 83% reduction in worm egg burden were obtained without the need for chemical adjuvants (Table 8) [62]. Cysteine and serine protease allergens cloned from other sources confirm this result providing evidence for peptidases to behave simultaneously as immunogens and adjuvants [60,109]. This offers an innovative approach towards vaccine development [62].

## 3. Perspectives and Conclusions

Adjuvant selection has a large impact on the effectiveness of the vaccine and the use of adjuvants to aid in the stimulation of the immune system is a critical step and a major variable affecting vaccine development. However, there is still a tendency to employ the few approved ones with the wrong intention to rapidly progress to clinical trials. Before adjuvant selection can be made, a comprehensive understanding of the immune system, level of protection and the desired immune response is required—or, more importantly, it is important to know which immune responses should be avoided. It is also essential to examine the type of immune response that the candidate vaccine antigen induces in the target host under natural infection without inclusion of any adjuvant [4]. For schistosomiasis, this is complicated as very few individuals develop natural resistance to reinfection with the schistosome parasite in the absence of repeated praziquantel treatment. Therefore, tailoring vaccine development to individuals is required, albeit impractical. Additionally, factors that affect the response of each antigen/adjuvant are also dependent, to some extent, on the animal model employed. For example, BALB/c and BL/6 mice are considered high responders to the vaccine made from irradiated *S. mansoni* cercariae with fewer worms observed after challenge infection than occurs with moderate responders, such as CBA mice. However, infected CBA mice show a stronger splenic proliferation response and a lesser suppressor T cell response once a schistosome infection becomes patent than do high responder mice. From this, the selection of mouse strain used in the vaccine/challenge model is an important aspect to consider in the experimental design and for critical interpretation/comparison of results [38]. Translational research from mouse to humans are complicated by disease severity and immune response variations in outbred populations and only a small number of studies have tried to compare these. To add to this, correlation of the mouse to human model is also under studied [4,110,111,112,113]. Here, detailed analysis, and genetic and immunological studies in mice and humans are required to identify a suitable model for schistosome vaccine development. Moreover, it is not clear what effect genetic susceptibility of individuals to make a Th1 response would have on their ability to subsequently develop resistance to infection, given that the latter seems to be Th2-responses-mediated in the endemic setting [4,110,111,112,113].

Traditionally, CFA is used when a candidate antigen is first being assessed as a vaccine and although it has been backbone of immunological adjuvants in research for decades, it is still not suitable for human applications. Therefore, once efficacy has been established with CFA, other adjuvants should be explored to formulate a vaccine antigen. Less conventional or less widely used approaches have been explored as adjuvants for schistosome vaccines, including cholera toxin, BCG, liposomes, and others (refer to Table 3, Table 4, Table 5, Table 6, Table 7 and Table 8 for details).

Several reasons have led to the widespread use of traditional adjuvants including cost, difficulty to access new adjuvants, formulation ease and characterisation. On the other hand, adjuvants are not approved as a product alone but in combination with a vaccine or formulation defining a determined combination of antigen(s) plus adjuvant(s) and each combination requires full product development, restraining the progression of those adjuvants for new vaccine applications. Fortunately, over the past few years, new rules to promote the use of new adjuvants have been initiated [6].

It is important to know how adjuvants work in order to determine their role in vaccine formulation and to design new adjuvants. Recent advances in parasitic disease and immune response pathways aid this. For instance, although Alum has been around for more than 80 years, only recently has insight into its mechanism of action been described. These steps have also promoted the discovery of new adjuvants [6]. Improved knowledge of toll-like and pattern recognition receptors and the link between innate and adaptive immunity has enabled a new generation of synthetic adjuvants overcoming some of the concerns with toxicity, potency, and manufacturing problems [6]. Olds *et al*., attempted to identify and correlate immune responses between 10 schistosomiasis vaccine candidates and to investigate their association with resistance *vs.* susceptibility to re-infection in human participants from Egypt [44]. Highly specific humoral and cellular immune reactions in response to the 10 antigens correlated, both prospectively and retrospectively, with detailed epidemiological data covering a 66-month period. Each antigen produced a unique immune response profile but no clear “winner(s)” was recognised. However, a marker for both resistance and susceptibility to re-infection was identified for each molecule indicating which types of responses to aim for in vaccination and which ones to avoid. Insights gained from this approach will be useful for antigen selection and ultimately for vaccine formulation prior to clinical trials in humans [44].

The bar to achieve protective efficacy in humans was set in the 1990s at a consistent induction of 40% protection or better by the World Health Organisation (WHO), and although this is a modest goal, it was not reached with the six most promising schistosomiasis vaccine candidates (Sm28GST, IrV5, Sm14, paramyosin, TPI, and Sm23) at the time [114]. This highlighted the need for standardised and effective adjuvant formulations.

All of this information is relevant in deciding how best to formulate and deliver a vaccine for schistosomiasis. Skewing an immune response towards Th1 is currently seen as the most promising way to obtain protection, and this can be achieved using Th1-driving adjuvants. Recent focus on the use of cytokines (e.g., IL-12 and IL-18) as adjuvants has been met with mixed results [8]. Lung stage antigen administered with IL-12 was shown to be a powerful inducer of a Th1 immune response, boosting protection levels to 90%. However, IL-12 failed to protect when administered with Sm28GST antigen. Furthermore, cytokines as adjuvants may not be very feasible with the high production cost and the logistics of the distribution and administration of the vaccine [8]. Here, formulations that minimise the need for refrigeration and avoid the use of needles are highly desirable. Adjuvants such as unmethylated CpG ODNs are also attractive, showing promise for experimental vaccines against other parasites by targeting the TLRs. Additionally, if a mixed Th1/Th2 response is desired, combination adjuvants such as alum-CpG seem to be an appropriate way forward. Finally, Lewis X-based carbohydrates isolated from schistosome eggs offer a promising antigen without the need for an additional adjuvant [4].

Vaccine-challenge experiments modifying the delivery of the vaccine and testing the different adjuvant formulation will enable a better assessment of an adjuvant’s role in inducing protective immunity. Furthermore, combination adjuvants are promising next generation adjuvants offering tailored immune responses to new antigen targets [115]. In addition, the efficacy of anti-schistosome vaccines could be successfully enhanced by combining new combinations of existing adjuvants with novel ones developed based on emerging immunological targets.
